# Within-Person Longitudinal Associations Between Parental Depressive Symptoms and Children’s Emotional and Behavioral Functioning: Differences by Household Income

**DOI:** 10.3390/children13101350

**Published:** 2026-10-05

**Authors:** Jiho Park

**Affiliations:** Department of Social Welfare, Seoul Women’s University, Seoul 01797, Republic of Korea; jhpark25@swu.ac.kr

**Keywords:** parental depressive symptoms, internalizing behaviors, externalizing behaviors, household income, random-intercept cross-lagged panel model

## Abstract

**Highlights:**

**What are the main findings?**

- Children’s higher-than-usual internalizing and externalizing behaviors predicted subsequent higher-than-usual parental depressive symptoms during the later elementary school years.
- Within-person reciprocal associations between parental depressive symptoms and children’s internalizing behaviors emerged only among lower-income families but not among relatively higher-income families.

**What are the implications of the main findings?**

- Children’s emotional and behavioral difficulties may signal broader family mental health needs, supporting attention to caregiver well-being when child difficulties are identified.
- Family-centered, two-generation approaches may be particularly relevant for economically disadvantaged families.

**Abstract:**

**Background/Objectives**: Parental depressive symptoms are consistently associated with children’s emotional and behavioral functioning, yet research has predominantly emphasized parent-to-child associations. Less is known about child-to-parent pathways and reciprocal processes, particularly across socioeconomic contexts. This study examined within-person reciprocal associations between parental depressive symptoms and children’s emotional and behavioral functioning across Grades 3–5 and tested whether these associations differed by household income status. **Methods**: Data were drawn from 5320 children (Mage = 9.10 years at Grade 3) in the Early Childhood Longitudinal Study, Kindergarten Class of 2010–11 (ECLS-K:2011), a nationally representative U.S. longitudinal study. Parental depressive symptoms and teacher-rated internalizing and externalizing behaviors were assessed annually. Random-intercept cross-lagged panel models (RI-CLPMs) examined within-person reciprocal associations, and multigroup RI-CLPMs tested income-group differences. **Results**: In the overall sample, parental depressive symptoms did not significantly predict subsequent internalizing or externalizing behaviors, whereas both child behaviors predicted subsequent parental depressive symptoms. Both the parent-to-child and child-to-parent pathways for internalizing behaviors differed significantly by household income status. Among lower-income families, parental depressive symptoms and children’s internalizing behaviors were reciprocally associated, whereas neither direction was significant among relatively higher-income families. In contrast, the cross-lagged associations for externalizing behaviors did not differ by household income status. **Conclusions**: Within-person reciprocal associations between parental depressive symptoms and children’s internalizing behaviors emerged among lower-income families but not among relatively higher-income families, suggesting that parent–child mental health processes may be more interconnected under socioeconomic disadvantage.

## 1. Introduction

Parental depressive symptoms are well established as being associated with children’s emotional and behavioral functioning, including internalizing and externalizing behaviors [1,2,3]. Emotional and behavioral difficulties during the school years may interfere with successful school adjustment and contribute to longer-term developmental difficulties extending into adulthood [4,5,6]. Given the established links between parental depressive symptoms and child adjustment, understanding how parent and child mental health are interconnected during this developmental period is therefore important.

Although a substantial body of research has predominantly focused on parent-to-child associations, growing evidence suggests that children’s emotional and behavioral functioning may also contribute to subsequent parental depressive symptoms [4,7,8]. Clarifying the direction of these associations is important for prevention and intervention, as such processes may call for approaches that address both parent and child mental health [4,9]. Yet comparatively little research has examined these reciprocal processes specifically during the later elementary school years using repeated longitudinal assessments to clarify how parent–child mental health dynamics unfold over time.

Against this background, the present study examined reciprocal associations between parental depressive symptoms and children’s emotional and behavioral functioning across the later elementary school years using data from a nationally representative U.S. longitudinal study. The study further examined whether these reciprocal associations differed by household income status.

### 1.1. Bidirectional Associations Between Parental Depressive Symptoms and Children’s Emotional and Behavioral Functioning

The possibility of influence in both directions is consistent with transactional models of development [10,11]. Rather than viewing children as passive recipients of parental characteristics and experiences, transactional perspectives conceptualize development as the product of continuing reciprocal exchanges between children and their caregiving environments [10,11]. In this framework, parental depressive symptoms may contribute to children’s emotional and behavioral difficulties, while children’s difficulties may in turn place additional psychological demands on parents and contribute to subsequent changes in parental well-being. This perspective therefore emphasizes the dynamic nature of parent–child mental health processes over time. More recent dyadic developmental perspectives similarly emphasize that parent and child emotional functioning is embedded within ongoing co-regulatory processes, in which parents and children mutually adapt to one another’s emotional and behavioral states [12]. A growing body of longitudinal research has provided support for this transactional perspective [4,7,13,14]. Some studies have reported reciprocal associations between parental depressive symptoms and children’s emotional and behavioral functioning, including internalizing and externalizing behaviors [4,7,9]. However, other longitudinal studies have identified asymmetric or developmentally specific patterns of association. For example, Daundasekara et al. [13] found that child anxiety/depressive symptoms predicted subsequent maternal depression across the period from preschool to adolescence, whereas maternal depression predicted subsequent child symptoms only from middle childhood to adolescence. Other studies have found parent-to-child associations without corresponding child-to-parent associations. Hentges et al. [15], for example, found that maternal depressive symptoms predicted subsequent child internalizing behaviors, whereas child internalizing behaviors did not predict later maternal depressive symptoms. Thus, although longitudinal research supports the possibility of influence in both directions, the direction and timing of prospective associations have varied across studies.

Importantly, findings regarding reciprocal associations may also depend on the analytic approach used to examine longitudinal change. Much of the earlier research relied on traditional cross-lagged panel models (CLPMs), which do not distinguish stable between-person differences from time-specific within-person variation. As a result, cross-lagged associations from these models may partly reflect enduring differences between families rather than prospective associations among time-specific within-person deviations. More recent studies have addressed this limitation using random-intercept cross-lagged panel models (RI-CLPMs), which separate stable between-person variance from time-specific within-person variation (e.g., [8,16]). Findings from this emerging literature have generally shown stable between-person associations linking parental depressive symptoms with children’s emotional and behavioral functioning, whereas prospective within-person associations have been mixed and often nonsignificant [17]. For example, Lowthian et al. [18] found a reciprocal within-person pattern for children’s emotional problems from middle childhood to early adolescence, but not for conduct problems. In contrast, Chad-Friedman et al. [19] found no reciprocal within-person associations between maternal depressive symptoms and child internalizing symptoms from ages 3 to 12, although a significant child-to-parent pathway emerged for irritability. Similarly, Feng et al. [17] found no reciprocal associations between parental depressive symptoms and children’s internalizing or externalizing behaviors in models simultaneously accounting for maternal and paternal depressive symptoms. Collectively, these findings indicate that prospective within-person associations remain inconsistent after stable between-person differences are separated.

Beyond these methodological limitations, several additional gaps remain in the existing literature. Much of the literature has relied on parents to report both their own depressive symptoms and their children’s behaviors, raising concerns about shared-informant variance [16,17,19,20]. Indeed, associations between maternal depression and child internalizing and externalizing behaviors have been stronger when mothers themselves reported child functioning than when other informants were used [2]. In addition, reciprocal research has often focused on early childhood [4,16] or broad developmental spans extending into adolescence [8,13,19], leaving comparatively limited evidence focused specifically on the later elementary school years. Thus, it remains unclear whether time-specific within-person deviations in parental depressive symptoms and children’s internalizing and externalizing behaviors prospectively predict one another during the later elementary school years.

### 1.2. The Role of Household Income in Parent–Child Mental Health Dynamics

Household economic conditions may shape not only levels of parent and child mental health difficulties but also the processes linking them over time. The family stress model proposes that economic hardship can increase parental psychological distress and disrupt family functioning, which may in turn contribute to children’s emotional and behavioral difficulties [21,22]. Extending this reasoning to reciprocal parent–child mental health processes, parents facing economic hardship may be more vulnerable to the demands associated with children’s emotional and behavioral difficulties, while children in economically strained households may likewise be more susceptible to difficulties associated with parental psychological distress [23]. Thus, household income may plausibly influence the strength of reciprocal parent–child mental health associations.

Consistent with this possibility, research in economically disadvantaged families has documented reciprocal associations between maternal depressive symptoms and children’s behavioral functioning (e.g., [7,24,25]). Other longitudinal work has also shown that poverty is associated with poorer mental health among both parents and children [26], suggesting that socioeconomic context may be relevant to these parent–child associations. However, prior studies have either focused exclusively on low-income families or examined poverty primarily as a predictor, rather than directly testing whether reciprocal associations differ across household-income groups. Moreover, some earlier studies relied on cross-lagged approaches that did not explicitly separate stable between-person differences from time-specific within-person variation (e.g., [7,25]).

To my knowledge, only one study has directly tested whether reciprocal parent–child mental health associations differ by household economic context. Using multigroup autoregressive latent trajectory models with structured residuals (ALT-SR), Saribaz et al. [23] found no evidence that household poverty moderated bidirectional associations between parental psychological distress and children’s internalizing and externalizing problems at either the between- or within-family level. Accordingly, further research is needed to determine whether income-group differences in reciprocal associations are evident when focusing specifically on parental depressive symptoms and independently reported child emotional and behavioral functioning during the later elementary-school years.

### 1.3. The Current Study

Building on these gaps, the present study examined reciprocal longitudinal associations between parental depressive symptoms and children’s emotional and behavioral functioning at the within-person level across Grades 3 through 5 using data from the Early Childhood Longitudinal Study, Kindergarten Class of 2010–11 (ECLS-K:2011). Children’s emotional and behavioral functioning was assessed by teachers, providing an independent source of information from parents’ reports of their own depressive symptoms. RI-CLPMs were used to separate stable between-person differences from time-specific within-person deviations and to estimate prospective associations in both parent-to-child and child-to-parent directions.

The study addressed two research questions (RQs): (1) To what extent are parental depressive symptoms and children’s emotional and behavioral functioning reciprocally associated at the within-person level? That is, to what extent do parental depressive symptoms at an earlier time point predict children’s internalizing and externalizing behaviors at a later time point, and vice versa? (2) To what extent do these reciprocal within-person associations vary by household income status? Given the mixed evidence regarding within-person reciprocal associations and the limited evidence concerning income-group differences, no directional hypotheses were specified.

## 2. Materials and Methods

### 2.1. Data and Sample

This study used three waves of data (the spring semesters of third, fourth, and fifth grades) from the public-use version of ECLS-K:2011. The ECLS-K:2011 was conducted by the National Center for Education Statistics (NCES), within the Institute of Education Sciences of the U.S. Department of Education. The ECLS-K:2011 is a nationally representative longitudinal study of U.S. children who entered kindergarten during the 2010–2011 school year and were followed through the spring of fifth grade in 2016. The study employed a multistage sampling design and initially included approximately 18,000 children from diverse racial, ethnic, and socioeconomic backgrounds who attended 1319 public and private schools across 90 geographic areas in the U.S. Across the elementary school years, NCES field staff directly assessed participating children and collected information from their parents, teachers, and school administrators.

The focal variables in the current study, parental depressive symptoms and children’s emotional and behavioral functioning, were assessed from third through fifth grade. From the initial ECLS-K:2011 cohort of approximately 18,000 children, the analytic sample was restricted to children with valid values on the longitudinal sampling weight (W9C19P_9T29B0). Children without a valid value for this weight were excluded. This weight was selected because it includes nonresponse adjustments for the parent and teacher data used in the present analyses across the spring of third, fourth, and fifth grades. Application of this sample-selection criterion resulted in a final analytic sample of 5320 children (Mage = 9.10 years in the third grade), of whom 49% were female; 60% were non-Hispanic White, 7% non-Hispanic Black/African American, 21% Hispanic, 6% Asian, and 6% other race/ethnicity. Mothers (biological mothers and other types) were the primary parent respondents (87.1%), followed by fathers (biological fathers and other types) (9.9%), grandparents or other relatives (2.9%), and other non-relative respondents (0.1%). Approximately 96% of the analytic sample had the same primary parent or caregiver respondent across Grades 3–5. The mean age of the primary parent reporter was 38.5 years when the child was in third grade. Children included in the analytic sample were more likely to be White, less likely to come from lower-income households, and slightly older than children excluded from the analytic sample.

### 2.2. Measures

#### 2.2.1. Parental Depressive Symptoms

Parental depressive symptoms were assessed in the third, fourth, and fifth grades using a 12-item short-form adaptation of the Center for Epidemiologic Studies Depression Scale (CES-D; [27]). Throughout the manuscript, parental depressive symptoms refer to depressive symptoms reported by the child’s primary parent or caregiver respondent. Parent or caregiver respondents reported how often during the past week they had experienced depressive symptoms (e.g., poor appetite, feeling fearful, feeling sad, and feeling unusually bothered) using a four-point scale ranging from 0 (never) to 3 (most of the time). Responses to the 12 items were summed to create a continuous score ranging from 0 to 36, with higher scores indicating greater depressive symptom severity. The scale demonstrated good internal consistency across third, fourth, and fifth grades (α = 0.85, 0.86, and 0.86, respectively).

#### 2.2.2. Children’s Emotional and Behavioral Functioning

Teacher-rated internalizing and externalizing behaviors were used to assess children’s emotional and behavioral functioning in the spring of third, fourth, and fifth grades. Items were adapted from the Social Skills Rating System (SSRS; [28]). Teachers rated how often each child exhibited specific behaviors using a four-point response scale ranging from 1 (never) to 4 (very often). Due to copyright restrictions, the ECLS-K:2011 does not provide the exact wording of all items but provides composite scores. Internalizing behaviors were assessed with four items reflecting anxiety, loneliness, sadness, and low self-esteem. Externalizing behaviors were assessed with six items reflecting arguing, fighting, impulsivity, anger, and disruption of ongoing activities. Higher scores indicated more frequent behaviors. Internal consistency across third through fifth grade ranged from α = 0.78 to 0.79 for internalizing behaviors and from α = 0.87 to 0.88 for externalizing behaviors [29].

#### 2.2.3. Household Income Status

Household income status was measured using the ECLS-K:2011 household poverty composite in the third grade. Parents were asked to report total household income for the previous year, first by broad range ($25,000 or less or more than $25,000) and then by 18 detailed income categories ($5000 or less to $200,001 or more). NCES constructed the household poverty status variable based on household income, household size, and the 2013 U.S. Census Bureau poverty thresholds. The household poverty measure included three categories: (1) below the poverty threshold, (2) at or above the poverty threshold but below 200% of the poverty threshold, or (3) at or above 200% of the poverty threshold. In the present study, categories 1 and 2 were combined to distinguish households below 200% of the poverty threshold (lower-income; coded as 1) from those at or above 200% (relatively higher-income; coded 0). This cutoff was selected to capture a broader range of economic disadvantage than the official poverty threshold alone and is consistent with prior research using ECLS-K:2011 and other child-focused studies that have operationalized lower-income status using this cutoff (e.g., [30,31]). Of the 5320 children in the analytic sample, 1963 (36.9%) were from lower-income households.

### 2.3. Analytic Plan

Descriptive statistics and bivariate correlations were first examined among the focal variables. Intraclass correlation coefficients (ICCs) were also estimated for parental depressive symptoms and children’s internalizing and externalizing behaviors across the three waves to assess the proportion of variance attributable to stable between-person differences and the extent of within-person variability over time.

Prior to the primary longitudinal analyses, measurement invariance of parental depressive symptoms was evaluated to determine whether the construct was measured equivalently over time and across family income groups. Configural, metric, and scalar invariance models were tested sequentially. Changes in model fit were evaluated using the following criteria: changes in the Comparative Fit Index (CFI) of ≤0.010, changes in the Root Mean Square Error of Approximation (RMSEA) of ≤0.015, and changes in the Standardized Root Mean Square Residual (SRMR) of ≤0.030 for metric invariance and ≤0.010 for scalar invariance [32]. Based on these criteria, scalar invariance was supported for parental depressive symptoms over time as well as across household income groups (see Supplementary Table S4). Given evidence of longitudinal measurement invariance and to maintain model parsimony, composite scores for parental depressive symptoms were used as observed variables in the subsequent RI-CLPM analyses rather than modeling depressive symptoms as latent factors.

To address RQ1, two RI-CLPMs were estimated to examine reciprocal associations between parental depressive symptoms and children’s internalizing and externalizing behaviors, respectively. All constructs (parental depressive symptoms and children’s internalizing and externalizing behaviors) were modeled as observed composite scores at each wave. The RI-CLPM decomposes repeated measures into stable between-person differences and time-specific within-person fluctuations by including a random intercept for each repeatedly assessed construct [33,34]. The random intercepts capture stable between-person differences in the average levels of each construct across measurement occasions, whereas the within-person components represent occasion-specific deviations from the expected level of each construct. At the within-person level, autoregressive and cross-lagged paths and concurrent residual covariances were estimated simultaneously. Autoregressive paths indicate whether deviations from one’s expected level persist to the subsequent occasion. Cross-lagged paths indicate whether a deviation in one construct predicts a subsequent deviation in the other construct. Concurrent residual covariances capture associations between within-person deviations occurring at the same measurement occasion. A fully constrained model, in which the autoregressive paths, cross-lagged paths, and concurrent residual covariances were constrained to equality across waves, was compared with a less constrained model in which these parameters were freely estimated across waves. Equality constraints were imposed on the unstandardized parameters, and standardized estimates are reported for interpretation. Nested models were compared using the Satorra–Bentler scaled chi-square difference test (ΔS–B χ^2^; [35]), with a significant difference indicating better fit for the less constrained model. Model fit was evaluated using the CFI, TLI, RMSEA, and SRMR. Values of CFI and TLI ≥ 0.95, SRMR ≤ 0.08, and RMSEA ≤ 0.06 were considered indicative of excellent fit, whereas CFI and TLI ≥ 0.90 and SRMR and RMSEA ≤ 0.08 indicated acceptable fit [36].

To address RQ2, multigroup RI-CLPMs were estimated separately for internalizing and externalizing behaviors. A fully constrained model was first estimated in which corresponding autoregressive paths, cross-lagged paths, and concurrent residual covariances were constrained to equality across income groups. These equality constraints were then selectively relaxed in a series of nested comparisons, beginning with the cross-lagged paths, to determine which sets of within-person parameters differed across groups. Nested models were compared using ΔS–B χ^2^, as described above, with a significant difference indicating improved fit for the less constrained model. Model fit was evaluated using the same criteria described above. As in the overall-sample models, equality constraints in the multigroup RI-CLPMs were applied to unstandardized parameters. When the omnibus comparison indicated significant income-group differences in the cross-lagged paths, follow-up Wald tests were conducted to determine whether each directional cross-lagged pathway differed significantly between groups.

All preliminary analyses were conducted using Stata 17.0, and all models were estimated in Mplus 8.3 [37]. The longitudinal sampling weight (W9C19P_9T29B0) was incorporated in all models to account for unequal probabilities of selection and for survey nonresponse associated with the response components used in constructing this weight. The use of this weight improves the generalizability of the findings to the national population of children who entered kindergarten in the United States during the 2010–2011 school year, subject to the eligibility and response criteria underlying the weight. Missing data in the analysis variables were handled using full information maximum likelihood (FIML; [38]). To account for the multistage sampling design of the ECLS-K:2011, the analyses also incorporated the corresponding stratification (W9C19P_9T29BSTR) and clustering variables (W9C19P_9T29BPSU) using the TYPE = COMPLEX command in Mplus. These design adjustments address the oversampling of selected population subgroups and the stratified cluster design, thereby producing standard errors and model test statistics that appropriately reflect the survey design. Robust maximum likelihood (MLR) estimation was used to account for the non-normality in the study variables.

Two different sensitivity analyses were conducted. First, the full-sample RI-CLPM and the multigroup RI-CLPMs were re-estimated using a sample restricted to children with the same parent or caregiver respondent across all three waves. Second, the multigroup RI-CLPMs were re-estimated using an alternative household income cutoff based on the official poverty threshold, comparing households below 100% of the poverty threshold with those at or above 100%.

## 3. Results

### 3.1. Preliminary Results

Table 1 presents unweighted and weighted descriptive statistics for the study variables for the full sample and by household income group (lower-income and relatively higher-income households). Across Grades 3–5, parental depressive symptoms and children’s internalizing and externalizing behaviors were consistently higher in lower-income households than in relatively higher-income households.

Weighted bivariate correlations for the full sample are presented in Table 2 (see Supplementary Table S1 for full parameter). Within-construct correlations across Grades 3–5 were moderate for parental depressive symptoms (0.539–0.568) and externalizing behaviors (0.544–0.602), and smaller for internalizing behaviors (0.324–0.378). Parental depressive symptoms showed small positive correlations with children’s internalizing (0.094–0.153) and externalizing behaviors (0.051–0.140). All correlations were statistically significant at *p* < 0.001. Bivariate correlations stratified by household income group are presented in Supplementary Tables S2 and S3. ICCs were 0.53 for parental depressive symptoms, 0.36 for internalizing behaviors, and 0.57 for externalizing behaviors. Thus, 47%, 64%, and 43% of the variance, respectively, was attributable to within-person fluctuations, indicating sufficient within-person variability to examine longitudinal within-person associations using RI-CLPMs.

### 3.2. RI-CLPMs of Parental Depressive Symptoms and Children’s Emotional and Behavioral Functioning

Both the fully constrained and fully unconstrained RI-CLPMs yielded fit indices within conventional thresholds for parental depressive symptoms with children’s internalizing and externalizing behaviors, respectively (see Supplementary Table S5). The fully constrained models did not significantly worsen model fit relative to the unconstrained models for either internalizing behaviors, ΔS–B χ^2^(5) = 3.27, *p* = 0.658, or externalizing behaviors, ΔS–B χ^2^(5) = 3.74, *p* = 0.588. Thus, the fully constrained models were retained for parsimony (internalizing behaviors: χ^2^(6) = 4.736, CFI = 1.000, TLI = 1.001, SRMR = 0.010, RMSEA = 0.000 [0.000, 0.016]; externalizing behaviors: χ^2^(6) = 7.363, CFI = 1.000, TLI = 0.999, SRMR = 0.013, RMSEA = 0.007 [0.000, 0.020]).

**Figure 1:**
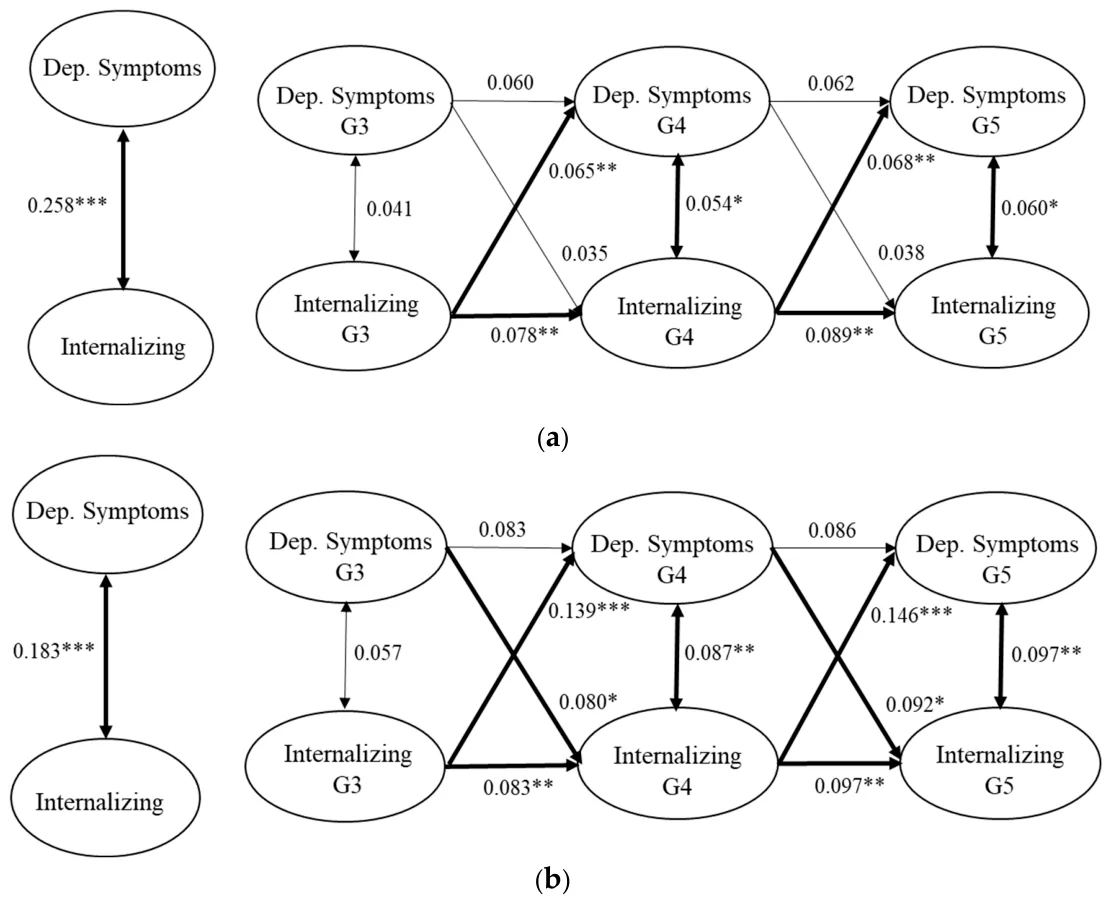

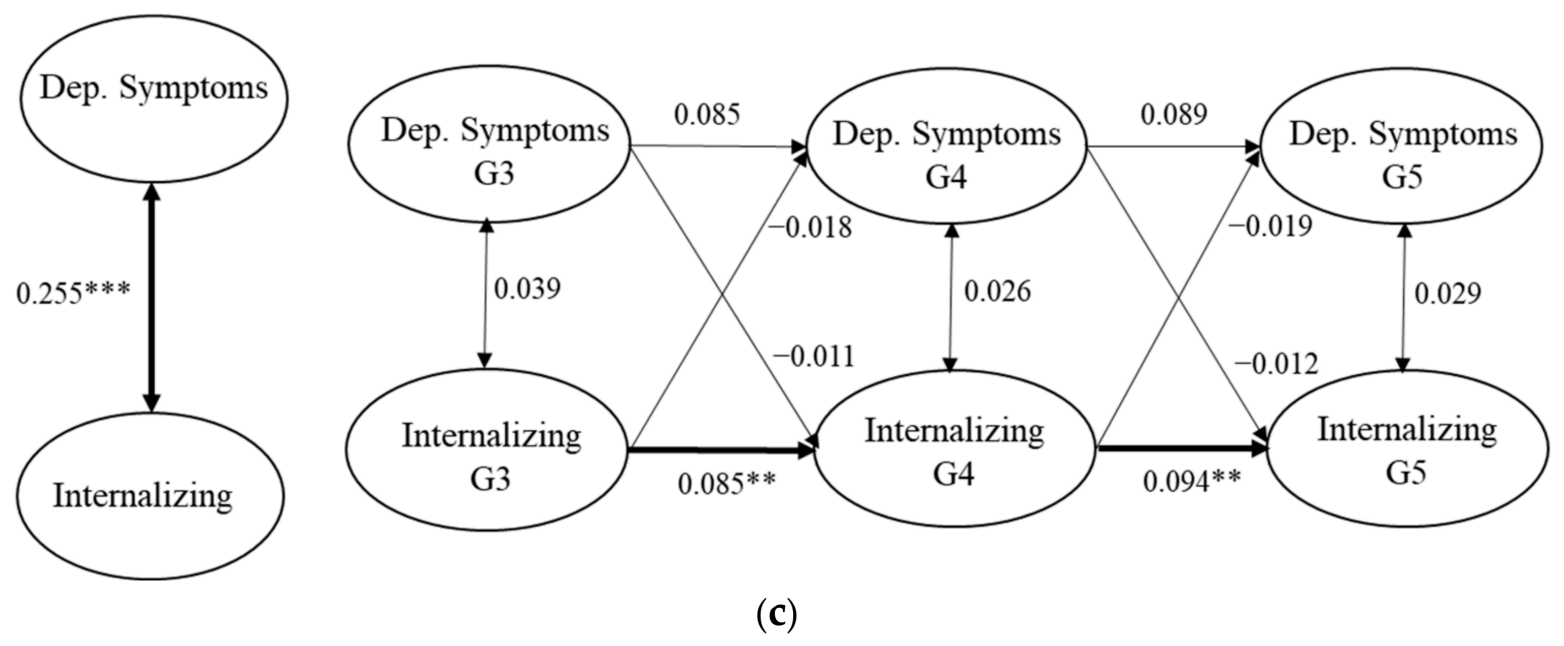
RI-CLPMs on depressive symptoms and internalizing behaviors: (**a**) Full sample. (**b**) Lower-income. (**c**) Relatively higher-income. Note. Standardized estimates reported. Bold lines indicate statistical significance. Dep. Symptoms = Depressive symptoms. * *p* < 0.05, ** *p* < 0.01, *** *p* < 0.001.

Figure 1a shows the results of the final RI-CLPM for parental depressive symptoms and children’s internalizing behaviors (see Supplementary Table S6 for full parameter estimates). The cross-lagged paths from parental depressive symptoms to internalizing behaviors were not significant (*βs* = 0.035–0.038, *ps* = 0.127–0.128). In contrast, the cross-lagged paths from internalizing behaviors to parental depressive symptoms were positive and significant across both intervals (*βs* = 0.065–0.068, *ps* = 0.005–0.008). Internalizing behaviors showed significant autoregressive paths across grades (*βs* = 0.078–0.089, *ps* = 0.002–0.003), whereas autoregressive paths for parental depressive symptoms were not significant (*βs* = 0.060–0.062, *ps* = 0.316–0.350). Within-person concurrent associations were significant at Grades 4 and 5 (*βs* = 0.054–0.060, *ps* = 0.015–0.016), but not at Grade 3 (*β* = 0.041, *p* = 0.175). At the between-person level, higher average parental depressive symptoms were positively associated with higher average levels of children’s internalizing behaviors across time (*φ* = 0.258, *p* < 0.001).

**Figure 2:**
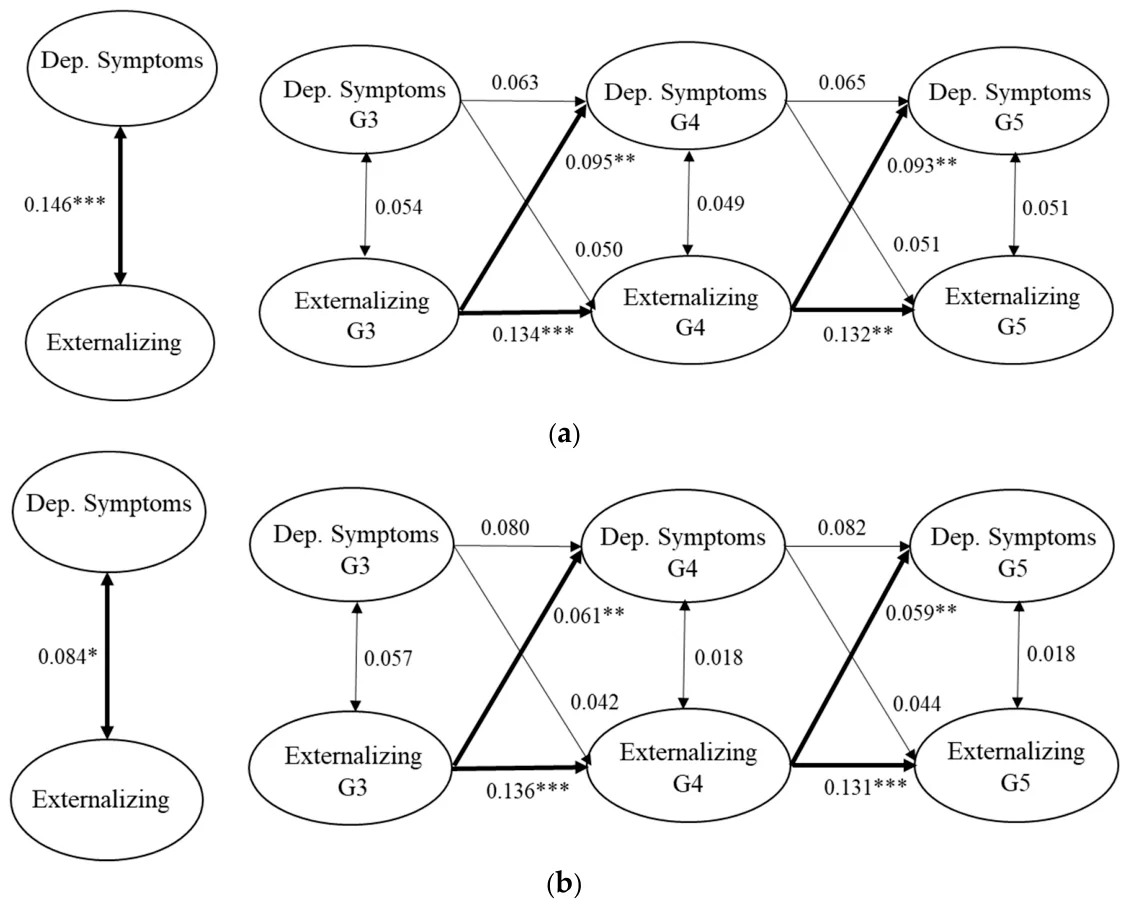

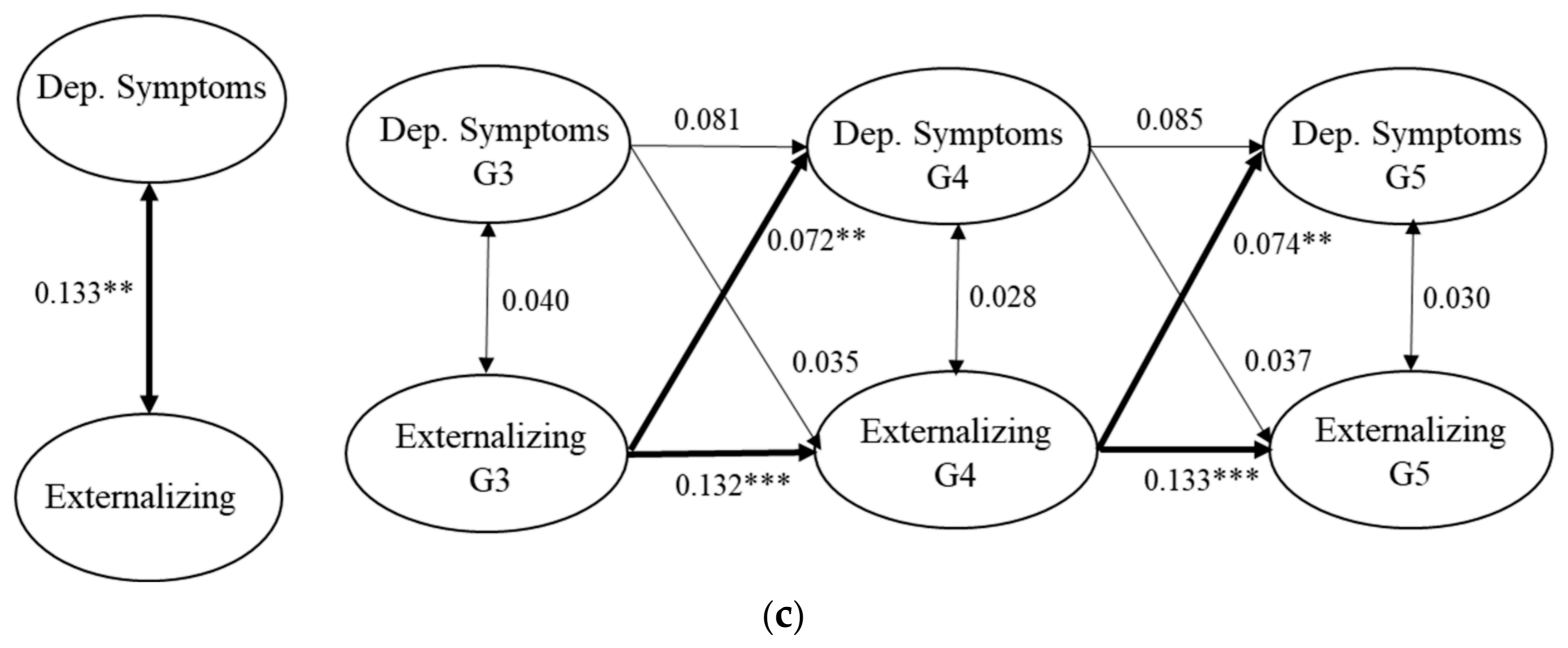
RI-CLPMs on depressive symptoms and externalizing behaviors: (**a**) Full sample. (**b**) Lower-income. (**c**) Relatively higher-income. Note. Standardized estimates reported. Bold lines indicate statistical significance. Dep. Symptoms = Depressive symptoms. * *p* < 0.05, ** *p* < 0.01, *** *p* < 0.001.

A similar pattern emerged for parental depressive symptoms and children’s externalizing behaviors (Figure 2a; Supplementary Table S7). The cross-lagged paths from parental depressive symptoms to externalizing behaviors were not significant (*βs* = 0.050–0.051, *ps* = 0.061–0.066), whereas the cross-lagged paths from externalizing behaviors to parental depressive symptoms were positive and significant across both intervals (*βs* = 0.093–0.095, *ps* = 0.002–0.003). All autoregressive paths for externalizing behaviors were significant (*βs* = 0.132–0.134, *ps* ≤ 0.001), but not for parental depressive symptoms (*βs* = 0.063–0.065, *ps* = 0.285–0.320). None of the within-person concurrent associations between parental depressive symptoms and externalizing behaviors were significant across the three grades (*ps* = 0.069–0.072). At the between-person level, higher average parental depressive symptoms were positively associated with higher average levels of children’s externalizing behaviors across time (*φ* = 0.146, *p* < 0.001).

### 3.3. Multigroup RI-CLPMs Across Household Income Groups

All multigroup RI-CLPMs showed good fit (see Supplementary Table S5). For internalizing behaviors, freeing the cross-lagged paths significantly improved model fit relative to the fully constrained model, ΔS–B χ^2^(2) = 12.49, *p* = 0.002. Additionally, freeing the concurrent residual covariances further improved fit, ΔS–B χ^2^(1) = 4.50, *p* = 0.034, whereas freeing the autoregressive paths did not. Thus, the model with cross-lagged paths and concurrent residual covariances freely estimated across income groups was retained (χ^2^(15) = 13.665, CFI = 1.000, TLI = 1.001, SRMR = 0.016, RMSEA = 0.000 [0.000, 0.017]). For externalizing behaviors, none of the sequentially relaxed models significantly improved fit relative to the fully constrained model; therefore, the fully constrained model was retained for parsimony (χ^2^(18) = 23.723, CFI = 0.998, TLI = 0.997, SRMR = 0.023, RMSEA = 0.011 [0.000, 0.022]).

For internalizing behaviors (Figure 1b,c; Supplementary Table S6), the cross-lagged associations differed by household income group. Among lower-income households, higher-than-usual parental depressive symptoms predicted subsequent higher-than-usual internalizing behaviors (*βs* = 0.080–0.092, *ps* = 0.012–0.014). Higher-than-usual internalizing behaviors also predicted subsequent higher-than-usual parental depressive symptoms (*βs* = 0.139–0.146, *ps* < 0.001), indicating reciprocal within-person associations. Among relatively higher-income households, neither the paths from parental depressive symptoms to internalizing behaviors (*βs* = −0.012 to −0.011, *ps* = 0.673–0.674) nor the reverse paths (*βs* = −0.019 to −0.018, *ps* = 0.469–0.472) were significant. Follow-up Wald tests confirmed that both directional cross-lagged paths differed significantly between income groups: the parent-to-child path, χ^2^(1) = 4.47, *p* = 0.034, and the child-to-parent path, χ^2^(1) = 15.81, *p* < 0.001.

For externalizing behaviors (Figure 2b,c; Supplementary Table S7), the cross-lagged associations did not significantly differ by household income group. Across income groups, externalizing behaviors significantly predicted subsequent parental depressive symptoms, whereas parental depressive symptoms did not significantly predict subsequent externalizing behaviors.

### 3.4. Sensitivity Analyses

Using a sample restricted to children with the same parent or caregiver respondent across all three waves, both the full-sample RI-CLPMs and the multigroup RI-CLPMs showed patterns similar to those of the primary analyses (see Supplementary Table S8). This analysis reduces concern that changes in the parent or caregiver respondent drove the primary findings, although it does not address other potential sources of informant-related measurement variation. When an alternative household income cutoff based on 100% of the poverty threshold was used, the overall pattern of findings was also similar, although the child internalizing-to-parent depressive symptoms associations were somewhat larger in the poverty group (see Supplementary Table S9).

## 4. Discussion

Although associations between parental depressive symptoms and children’s emotional and behavioral functioning are well established, less is known about their within-person reciprocal dynamics during the later elementary school years and whether these associations differ by household income status. The present study addressed these questions using three waves of data from a nationally representative U.S. longitudinal study and RI-CLPMs that separated stable between-person differences from time-specific within-person deviations. The study did not find evidence of within-person reciprocal associations between parental depressive symptoms and children’s internalizing or externalizing behaviors in the overall sample (RQ1); rather, significant unidirectional child-to-parent associations emerged for both behavioral domains, whereas the corresponding parent-to-child associations were not significant. Household income differentiated the cross-lagged associations for internalizing behaviors (RQ2). Within-person reciprocal associations emerged among lower-income families but not among relatively higher-income families. Follow-up Wald testing further confirmed significant between-group differences in both the parent-to-child and child-to-parent pathways. In contrast, the cross-lagged associations for externalizing behaviors did not differ by household income status.

Regarding RQ1, the findings provided qualified support for the transactional model of development. Higher-than-usual levels of children’s internalizing and externalizing behaviors predicted subsequent higher-than-usual parental depressive symptoms, consistent with the transactional view that children are active contributors to family processes rather than passive recipients of parental influence [10,11]. The asymmetric overall pattern is also broadly consistent with transactional perspectives suggesting that the relative strength and direction of parent–child processes may vary across developmental periods and family contexts [39].

The significant child-to-parent associations may reflect the psychological and caregiving demands associated with children’s emotional and behavioral difficulties. Such difficulties may increase child-related stress, worry, or self-blame among parents, which may subsequently contribute to greater depressive symptoms [7,40], although these potential psychological and caregiving processes were not directly assessed in the present study. Prior longitudinal studies have similarly documented prospective child-driven associations between children’s difficulties and later parental depressive symptoms or psychological distress [7,18,24]. The present findings are consistent with this pattern during the later elementary school years and across both internalizing and externalizing domains, suggesting that children’s emotional and behavioral difficulties may have implications not only for children themselves but also for subsequent parental well-being.

In contrast, in the overall sample, the corresponding parent-to-child coefficients were positive but did not reach statistical significance. This pattern differs from some earlier longitudinal studies using traditional cross-lagged or related models that reported parent-to-child or reciprocal associations between parental depressive symptoms and children’s functioning [4,7,9,13]. However, recent RI-CLPM studies have generally found more consistent between-person associations and more selective within-person paths [8,16,17,18,19]. In the present study, significant between-person associations similarly indicated that parents with higher average depressive symptoms tended to have children with higher average levels of both internalizing and externalizing behaviors. Once stable between-person differences were separated from time-specific within-person deviations, however, parental depressive symptoms did not significantly predict subsequent internalizing or externalizing behaviors in the overall sample.

Developmental timing may be one explanation for the nonsignificant parent-to-child paths. In a meta-analysis, Goodman et al. [2] found that associations between maternal depression and children’s internalizing and externalizing problems were stronger among younger children. Consistent with this age-related pattern, several longitudinal studies reporting parent-to-child or reciprocal associations focused on early childhood [4,7,9]. Findings across broader age ranges further suggest that such associations may emerge only during particular developmental periods [13]. Cioffi et al. [16] similarly found that maternal depressive symptoms at 18 months predicted child internalizing behavior at 27 months, with no other significant parent-to-child paths through age 6. Thus, the nonsignificant parent-to-child paths during Grades 3–5 may partly reflect developmental variation in these associations.

Finally, differences in informants may have contributed to the relatively weak parent-to-child associations. Children’s internalizing and externalizing behaviors in the present study were rated by teachers, whereas many previous studies relied on mothers to report both their own depressive symptoms and their children’s behavior (e.g., [9,24]). Goodman et al. [2] found significantly stronger associations between maternal depression and child functioning when child outcomes were reported by mothers rather than by other informants, such as teachers. The use of teacher reports therefore reduces concerns about shared-informant variance but may also yield weaker associations because teachers observe children primarily in the school context.

Regarding RQ2, the within-person reciprocal associations between parental depressive symptoms and children’s internalizing behaviors differed by household income status. Consistent with the direct between-group differences identified by the Wald tests, the group-specific estimates showed that among lower-income families, higher-than-usual parental depressive symptoms predicted subsequent higher-than-usual child internalizing behaviors and vice versa, indicating a reciprocal pattern. Neither association was significant among relatively higher-income families. This pattern suggests a reciprocal longitudinal association among lower-income families, whereby elevations in parental depressive symptoms and children’s internalizing behaviors were followed by subsequent elevations in one another over time. Recent dyadic perspectives suggest that parent–child dynamics may also involve neurocognitive and psychophysiological processes [41]. Although these processes were not assessed in the present study, future research incorporating such perspectives could help clarify the processes contributing to reciprocal parent–child associations over time. The emergence of this pattern among lower-income families is consistent with the family stress model, which proposes that economic strain increases psychological distress and disrupts family functioning [21,22]. Economic strain may heighten vulnerability in both directions. For parents, constrained financial and psychosocial resources may make the worry and caregiving demands associated with children’s internalizing difficulties more difficult to manage, while children may be more vulnerable to parental depressive symptoms when families are simultaneously managing chronic economic stress. In this way, economic hardship may reduce families’ capacity to buffer one another’s emotional difficulties, thereby strengthening reciprocal associations over time [23]. Consistent with this interpretation, Goodman et al. [2] found stronger associations between maternal depression and children’s internalizing problems in studies of low-income families than in studies of middle-, higher-, or mixed-income families. However, the income-specific reciprocal associations should be interpreted in light of the potential limitations of teacher-rated internalizing behaviors, which may be less observable in school settings and susceptible to reporter-specific variation across grades.

The income-specific pattern for internalizing behaviors differs from Saribaz et al. [23], who found no evidence that poverty moderated reciprocal within-family associations between parental psychological distress and children’s internalizing or externalizing problems. One possible explanation is that they assessed poverty when children were 9 months old, whereas parent–child mental health associations were examined from ages 3 to 17. In contrast, the present study assessed household income at Grade 3, closer to the period modeled. Further research is needed to clarify when household income moderates reciprocal parent–child mental health associations.

In contrast to internalizing behaviors, the cross-lagged associations between parental depressive symptoms and children’s externalizing behaviors did not differ by household income status. Consistent with the overall-sample findings, parental depressive symptoms did not significantly predict subsequent externalizing behaviors, whereas externalizing behaviors predicted subsequent parental depressive symptoms. Externalizing behaviors, such as arguing, aggression, or impulsivity, may create immediate and observable caregiving and disciplinary demands that contribute to parental distress. This may help explain why the child-to-parent association was evident regardless of household income, although this interpretation remains tentative because these caregiving processes were not directly assessed.

It should be noted that, although statistically significant, the observed cross-lagged associations were small in magnitude in the overall sample (*βs* = 0.065–0.095) and remained small to modest among lower-income families (*βs* = 0.059–0.146). Given the large sample size, statistical significance should be interpreted cautiously and should not be assumed to indicate practical or clinical importance.

### 4.1. Strengths and Limitations

This study has several strengths. First, the use of a large longitudinal sample drawn from a nationally representative study, together with sampling weights and adjustment for complex survey design, enhances the generalizability of the findings and produces less biased estimates. Second, the RI-CLPMs separated stable between-person differences from time-specific within-person deviations, allowing the study to estimate prospective within-person associations rather than conflating them with between-person differences, as can occur in traditional CLPMs [33]. Third, children’s internalizing and externalizing behaviors were rated by teachers, reducing concerns about shared-informant variance with parental reports of depressive symptoms. Finally, the multigroup analyses directly tested whether these within-person associations differed by household income, addressing a question that has received limited empirical attention.

Several limitations should be considered. First, although the longitudinal RI-CLPM design allows examination of temporal ordering, the observational data do not permit causal conclusions, and unmeasured time-varying factors may contribute to the observed associations. Future studies incorporating additional time-varying family and contextual factors may help clarify these processes. Second, assessments were conducted annually and were limited to Grades 3 through 5. More frequent assessments and studies spanning broader developmental periods could determine whether within-person reciprocal associations vary across shorter intervals or developmental transitions. In addition, because the RI-CLPMs were estimated using only three waves and some models had relatively few degrees of freedom, the near-perfect global fit indices should be interpreted cautiously and provide only limited evidence regarding the adequacy of the longitudinal processes represented by the models. Third, household income was assessed at Grade 3 and treated as a fixed dichotomous indicator, although families’ economic circumstances may change over time. In addition, household income may not fully capture other dimensions of socioeconomic adversity, such as material hardship, housing instability, parental employment, and access to social support. Future research should consider time-varying income and more multidimensional indicators of economic hardship. Fourth, parent respondents were predominantly mothers but also included fathers and other caregivers, precluding examination of potentially distinct maternal and paternal processes. Accordingly, the findings primarily reflect maternal depressive symptoms and may not generalize to paternal or other caregiver processes. Although approximately 96% of children had the same parent or caregiver respondent across Grades 3–5, a small proportion had a change in respondent across waves. Such changes could introduce respondent-specific variation that may not reflect true within-person changes in parental depressive symptoms. However, sensitivity analyses restricted to children with the same parent or caregiver respondent across all three waves yielded substantively similar findings, with the direction and statistical significance of the cross-lagged associations remaining consistent. Future studies that assess mothers and fathers separately and maintain consistent parent or caregiver respondents across repeated assessments may provide a more differentiated understanding of parent–child dynamics. Fifth, children may have been rated by different teachers across grades, potentially introducing reporter-specific variation into the repeated assessments. Such variation may have attenuated the stability of teacher-rated behaviors across waves and could also have affected the estimation of longitudinal associations, particularly for internalizing behaviors. Internalizing behaviors, such as anxiety, sadness, and loneliness, are less directly observable in the school setting than externalizing behaviors and may therefore be more susceptible to differences in teachers’ perceptions and ratings. Future studies incorporating multiple informants, including children themselves, would provide a more comprehensive assessment of children’s functioning.

Finally, children’s internalizing and externalizing behaviors were available only as composite scores in the public-use data, so measurement invariance of these constructs could not be evaluated, limiting the ability to establish measurement comparability across time and household income groups. Although the indicators comprising these composites—for example, anxiety and sadness within internalizing behaviors and fighting and impulsivity within externalizing behaviors—may reflect partly distinct developmental processes, the use of composite scores precluded examination of whether individual dimensions exhibited different longitudinal associations with parental depressive symptoms [19].

### 4.2. Implications

The findings may inform child and family mental health practice. The significant child-to-parent associations suggest that children’s emotional and behavioral difficulties may have implications not only for their own functioning but also for parental well-being. Accordingly, when children show elevated levels of internalizing or externalizing difficulties, attention to caregivers’ mental health, particularly depressive symptoms, may also be warranted, consistent with prior recommendations for family-centered assessment and intervention [9,42]. Because the present study was observational, however, these associations do not establish that changes in child functioning lead to changes in caregiver depressive symptoms, or vice versa. Rather, they suggest the potential value of considering child and caregiver functioning together in practical and clinical settings. Importantly, the findings suggest that socioeconomic context may shape how parent and child mental health difficulties are interconnected, indicating that family-centered and two-generation perspectives may be particularly relevant for economically disadvantaged families. In such contexts, family-centered approaches may be better informed by considering the economic stressors that surround parent and child mental health difficulties. This broader perspective is also consistent with emerging intervention research showing concurrent improvements in child behavior and caregiver mental health [43]. These implications may be particularly important during the later elementary-school years, when emotional and behavioral difficulties can interfere with school adjustment and contribute to longer-term developmental difficulties [4,5,6]. Thus, child-serving settings, including schools and mental health services, may provide opportunities to recognize children’s difficulties while also considering caregiver well-being and the broader socioeconomic circumstances in which families are functioning.

## Figures and Tables

**Table 1:** Descriptive statistics for full sample and by household income group.

	Full Sample (*N* = 5320)		Lower-Income (*n* = 1963)		Relatively Higher-Income (*n* = 3357)			
Variable	Unweighted Mean (SD)	Weighted Mean (SD)	Unweighted Mean (SD)	Weighted Mean (SD)	Unweighted Mean (SD)	Weighted Mean (SD)	Range	Missing (%)
Depressive symptoms								
Grade 3	4.09 (4.58)	4.43 (4.87)	5.10 (5.46)	5.31 (5.57)	3.53 (3.91)	3.71 (4.08)	0–36	5.00
Grade 4	3.88 (4.52)	4.24 (4.92)	4.82 (5.45)	5.15 (5.64)	3.36 (3.81)	3.50 (4.09)	0–36	4.77
Grade 5	3.65 (4.48)	3.91 (4.90)	4.42 (5.40)	4.65 (5.81)	3.23 (3.82)	3.32 (3.93)	0–36	6.92
Internalizing behaviors								
Grade 3	1.57 (0.51)	1.59 (0.52)	1.63 (0.55)	1.63 (0.56)	1.53 (0.49)	1.55 (0.49)	1–4	0.68
Grade 4	1.56 (0.53)	1.58 (0.54)	1.63 (0.57)	1.64 (0.58)	1.53 (0.50)	1.54 (0.50)	1–4	1.30
Grade 5	1.55 (0.50)	1.56 (0.50)	1.61 (0.53)	1.61 (0.53)	1.51 (0.48)	1.52 (0.48)	1–4	1.43
Externalizing behaviors								
Grade 3	1.63 (0.59)	1.67 (0.61)	1.69 (0.63)	1.74 (0.65)	1.59 (0.56)	1.61 (0.56)	1–4	0.41
Grade 4	1.60 (0.57)	1.65 (0.60)	1.67 (0.62)	1.71 (0.64)	1.56 (0.54)	1.59 (0.55)	1–4	0.88
Grade 5	1.58 (0.56)	1.63 (0.58)	1.66 (0.61)	1.70 (0.62)	1.54 (0.53)	1.57 (0.54)	1–4	0.86

Note. Missingness is based on the full sample. SD = standard deviation.

**Table 2:** Weighted bivariate correlation matrix for full sample.

Variable	(1)	(2)	(3)	(4)	(5)	(6)	(7)	(8)	(9)
(1) Depressive symptoms G3	1.000								
(2) Depressive symptoms G4	0.558	1.000							
(3) Depressive symptoms G5	0.539	0.568	1.000						
(4) Internalizing behaviors G3	0.132	0.143	0.094	1.000					
(5) Internalizing behaviors G4	0.138	0.139	0.141	0.378	1.000				
(6) Internalizing behaviors G5	0.128	0.130	0.153	0.324	0.356	1.000			
(7) Externalizing behaviors G3	0.104	0.103	0.051	0.310	0.167	0.154	1.000		
(8) Externalizing behaviors G4	0.140	0.121	0.127	0.173	0.289	0.169	0.602	1.000	
(9) Externalizing behaviors G5	0.110	0.096	0.095	0.154	0.142	0.277	0.544	0.599	1.000

Note. G = grade. All coefficients are significant at *p* < 0.001.

## Data Availability

The data presented in this study are available from the National Center for Education Statistics (NCES), Early Childhood Longitudinal Studies (ECLS) Data Products at https://nces.ed.gov/ecls/ accessed on 1 July 2026, reference number NCES 2019-050. These data were derived from the following resources available in the public domain: https://nces.ed.gov/ecls/dataproducts.asp#K-5 accessed on 1 July 2026.

## Supplementary Materials

The following supporting information can be downloaded at: https://www.mdpi.com/article/10.3390/children13101350/s1, Table S1. Weighted Bivariate Correlation Matrix for Full Sample, Table S2. Weighted Bivariate Correlation Matrix for Lower-Income, Table S3. Weighted Bivariate Correlation Matrix for Relatively Higher-Income, Table S4. Measurement Invariance for Depressive Symptoms, Table S5. Model Fit Statistics for RI-CLPMs, Table S6. RI-CLPMs on Depressive Symptoms and Internalizing Behaviors, Table S7. RI-CLPMs on Depressive Symptoms and Externalizing Behaviors, Table S8. Sensitivity Analyses Restricted to Children with the Same Respondent across Grades 3–5, Table S9. Sensitivity Analyses Using 100% of the Poverty Threshold.
