# Supplementary material for: Within-Person Longitudinal Associations Between Parental Depressive Symptoms and Children’s Emotional and Behavioral Functioning: Differences by Household Income
Source: Children (Basel). 2026 Oct 5;13(10):1350. doi: 10.3390/children13101350 (PMC13703283; doi:10.3390/children13101350)
Supplement: Supplementary file 1 [file children-13-01350-s001.zip › children-4581534-supplementary.pdf]

Supplementary Material

Table S1. Weighted Bivariate Correlation Matrix for Full Sample

Table S2. Weighted Bivariate Correlation Matrix for Lower-Income

Table S3. Weighted Bivariate Correlation Matrix for Relatively Higher-Income

Table S4. Measurement Invariance for Depressive Symptoms

Table S5. Model Fit Statistics for RI-CLPMs

Table S6. RI-CLPMs on Depressive Symptoms and Internalizing Behaviors

Table S7. RI-CLPMs on Depressive Symptoms and Externalizing Behaviors

Table S8. Sensitivity Analyses Restricted to Children with the Same Respondent across Grades 3–5

Table S9. Sensitivity Analyses Using 100% of the Poverty Threshold

Table S1. Weighted Bivariate Correlation Matrix for Full Sample

Variable	(1)	(2)	(3)	(4)	(5)	(6)	(7)	(8)	(9)
(1) Depressive symptoms G3	1.000								
(2) Depressive symptoms G4	0.558 (.020)	1.000							
(3) Depressive symptoms G5	0.539 (.022)	0.568 (.019)	1.000						
(4) Internalizing behaviors G3	0.132 (.017)	0.143 (.019)	0.094 (.020)	1.000					
(5) Internalizing behaviors G4	0.138 (.018)	0.139 (.018)	0.141 (.020)	0.378 (.015)	1.000				
(6) Internalizing behaviors G5	0.128 (.016)	0.130 (.018)	0.153 (.015)	0.324 (.015)	0.356 (.020)	1.000			
(7) Externalizing behaviors G3	0.104 (.024)	0.103 (.024)	0.051 (.014)	0.310 (.020)	0.167 (.020)	0.154 (.021)	1.000		
(8) Externalizing behaviors G4	0.140 (.019)	0.121 (.020)	0.127 (.017)	0.173 (.021)	0.289 (.020)	0.169 (.018)	0.602 (.015)	1.000	
(9) Externalizing behaviors G5	0.110 (.020)	0.096 (.021)	0.095 (.018)	0.154 (.018)	0.142 (.021)	0.277 (.021)	0.544 (.017)	0.599 (.017)	1.000

Note. G = grade. Standard errors are in parentheses. All coefficients are significant at $p < .001$.

Table S2. Weighted Bivariate Correlation Matrix for Lower-Income

Variable	(1)	(2)	(3)	(4)	(5)	(6)	(7)	(8)	(9)
(1) Depressive symptoms G3	1.000								
(2) Depressive symptoms G4	0.531 (.033)	1.000							
(3) Depressive symptoms G5	0.536 (.030)	0.562 (.033)	1.000						
(4) Internalizing behaviors G3	0.106 (.026)	0.146 (.029)	0.080 (.031)	1.000					
(5) Internalizing behaviors G4	0.135 (.037)	0.128 (.026)	0.166 (.029)	0.357 (.027)	1.000				
(6) Internalizing behaviors G5	0.124 (.025)	0.144 (.029)	0.163 (.029)	0.293 (.024)	0.309 (.033)	1.000			
(7) Externalizing behaviors G3	0.092 (.035)	0.084 (.031)	0.037 (.027)	0.322 (.033)	0.142 (.029)	0.117 (.029)	1.000		
(8) Externalizing behaviors G4	0.142 (.029)	0.112 (.031)	0.143 (.034)	0.172 (.036)	0.256 (.030)	0.114 (.025)	0.598 (.021)	1.000	
(9) Externalizing behaviors G5	0.107 (.029)	0.080 (.036)	0.113 (.031)	0.149 (.031)	0.096 (.027)	0.218 (.030)	0.530 (.024)	0.592 (.023)	1.000

Note. G = grade. Standard errors are in parentheses. All coefficients were significant at $p < .05$, except for the correlation between externalizing behaviors G3 and depressive symptoms G5.

Table S3. Weighted Bivariate Correlation Matrix for Relatively Higher-Income

Variable	(1)	(2)	(3)	(4)	(5)	(6)	(7)	(8)	(9)
(1) Depressive symptoms G3	1.000								
(2) Depressive symptoms G4	0.564 (.035)	1.000							
(3) Depressive symptoms G5	0.510 (.030)	0.542 (.028)	1.000						
(4) Internalizing behaviors G3	0.132 (.021)	0.109 (.022)	0.091 (.021)	1.000					
(5) Internalizing behaviors G4	0.108 (.026)	0.118 (.029)	0.075 (.027)	0.391 (.018)	1.000				
(6) Internalizing behaviors G5	0.106 (.028)	0.091 (.036)	0.106 (.024)	0.346 (.018)	0.395 (.020)	1.000			
(7) Externalizing behaviors G3	0.083 (.029)	0.096 (.036)	0.051 (.023)	0.283 (.026)	0.176 (.024)	0.179 (.027)	1.000		
(8) Externalizing behaviors G4	0.093 (.034)	0.099 (.045)	0.082 (.027)	0.162 (.025)	0.310 (.028)	0.203 (.029)	0.597 (.019)	1.000	
(9) Externalizing behaviors G5	0.068 (.034)	0.084 (.042)	0.044 (.026)	0.146 (.021)	0.171 (.028)	0.325 (.025)	0.548 (.021)	0.595 (.021)	1.000

Note. G = grade. Standard errors are in parentheses. All coefficients are significant at $p < .05$, except for the correlation between externalizing behaviors G3 and depressive symptoms G5.

Table S4. Measurement Invariance for Depressive Symptoms

Model	*χ2*	*df*	CFI	TLI	SRMR	RMSEA [90% CI]	Δ CFI	Δ SRMR	Δ RMSEA
Full sample									
1. Configural invariance	1607.390	555	0.947	0.940	0.039	0.019 [0.018, 0.020]			
2. Metric invariance	1577.800	577	0.950	0.945	0.040	0.018 [0.017, 0.019]	0.003	0.001	0.001
3. Scalar invariance	1632.595	599	0.948	0.946	0.040	0.018 [0.017, 0.019]	0.002	0.000	0.000
Across household income									
1. Configural invariance	3068.603	1182	0.916	0.910	0.101	0.025 [0.024, 0.026]			
2. Metric invariance	2947.418	1202	0.922	0.919	0.068	0.023 [0.022, 0.025]	0.006	0.033	0.002
3. Scalar invariance	3020.334	1224	0.920	0.918	0.069	0.024 [0.023, 0.025]	0.002	0.001	0.001

Note. CFI = Comparative Fit Index; TLI = Tucker–Lewis Index; SRMR = Standardized Root Mean Square Residual; RMSEA = Root Mean Square Error of Approximation; CI = Confidence Interval.

Table S5. Model Fit Statistics for RI-CLPMs

Model	Autoregressive	Concurrent covariance	Cross-lagged	χ2 (df)	CFI	TLI	SRMR	RMSEA [90% CI]	CM	S-B Δχ2	Δ df	p-value
**Full sample**												
Internalizing behaviors												
**1.1.**	Constrained	Constrained	Constrained	4.736 (6)	1.000	1.001	0.010	0.000 [0.000, 0.016]				
1.2	Free	Free	Free	1.894 (1)	1.000	0.994	0.005	0.013 [0.000, 0.041]	1.1	3.27	5	0.658
Externalizing behaviors												
**2.1**	Constrained	Constrained	Constrained	7.363 (6)	1.000	0.999	0.013	0.007 [0.000, 0.020]				
2.2	Free	Free	Free	5.379 (1)	0.999	0.980	0.029	0.007 [0.000, 0.054]	2.1	3.74	5	0.588
**Multigroup (lower-income vs. relatively higher-income)**												
Internalizing behaviors												
3.1	Constrained	Constrained	Constrained	28.038 (18)	0.996	0.993	0.027	0.014 [0.000, 0.024]				
3.2	Constrained	Constrained	Free	17.326 (16)	0.999	0.999	0.020	0.006 [0.000, 0.019]	3.1	12.49	2	0.002
**3.3**	Constrained	Free	Free	13.665 (15)	1.000	1.001	0.016	0.000 [0.000, 0.017]	3.2	4.50	1	0.034
3.4	Free	Constrained	Free	16.636 (14)	0.999	0.998	0.020	0.008 [0.000, 0.021]	3.2	1.03	2	0.597
Externalizing behaviors												
**4.1**	Constrained	Constrained	Constrained	23.723 (18)	0.998	0.997	0.023	0.011 [0.000, 0.022]				
4.2	Constrained	Constrained	Free	21.294 (16)	0.999	0.997	0.022	0.011 [0.000, 0.022]	4.1	2.39	2	0.303
4.3	Constrained	Free	Constrained	22.036 (17)	0.999	0.998	0.022	0.011 [0.000, 0.022]	4.1	1.80	1	0.179
4.4	Free	Constrained	Constrained	22.969 (16)	0.998	0.997	0.023	0.013 [0.000, 0.024]	4.1	1.15	2	0.562

Note. CFI = Comparative Fit Index; TLI = Tucker–Lewis Index; SRMR = Standardized Root Mean Square Residual; RMSEA = Root Mean Square Error of Approximation; CI = Confidence Interval; CM = Comparison Model; S-B Δχ2 = Satorra–Bentler scaled chi-square difference test. Bolded model numbers indicate the retained models.

Table S6. RI-CLPMs on Depressive Symptoms and Internalizing Behaviors

	Full sample (N = 5,320)				Lower-income (n = 1,963)				Relatively higher-income (n = 3,357)			
Path	*β*	*SE*	95% CI	*p-value*	*β*	*SE*	95% CI	*p-value*	*β*	*SE*	95% CI	*p-*value
Autoregressive Paths												
Depressive symptoms												
Grade 3 → Grade 4	0.060	0.060	[-0.058, 0.178]	0.316	0.083	0.046	[-0.008, 0.174]	0.074	0.085	0.048	[-0.010, 0.179]	0.080
Grade 4 → Grade 5	0.062	0.066	[-0.068, 0.191]	0.350	0.086	0.052	[-0.017, 0.189]	0.102	0.089	0.055	[-0.019, 0.198]	0.106
Internalizing behavior												
Grade 3 → Grade 4	0.078	0.026	[0.028, 0.129]	**0.002**	0.083	0.025	[0.034, 0.133]	**0.001**	0.085	0.025	[0.036, 0.134]	**0.001**
Grade 4 → Grade 5	0.089	0.030	[0.031, 0.147]	**0.003**	0.097	0.029	[0.040, 0.154]	**0.001**	0.094	0.029	[0.037, 0.150]	**0.001**
Cross-lagged Paths												
Depressive symptoms to Internalizing												
Grade 3 → Grade 4	0.035	0.023	[-0.010, 0.080]	0.128	0.080	0.033	[0.016, 0.144]	**0.014**	-0.011	0.026	[-0.063, 0.041]	0.673
Grade 4 → Grade 5	0.038	0.025	[-0.011, 0.088]	0.127	0.092	0.037	[0.020, 0.163]	**0.012**	-0.012	0.029	[-0.068, 0.044]	0.674
Internalizing to Depressive symptoms												
Grade 3 → Grade 4	0.065	0.024	[0.017, 0.112]	**0.008**	0.139	0.036	[0.069, 0.208]	**<.001**	-0.018	0.024	[-0.065, 0.030]	0.469
Grade 4 → Grade 5	0.068	0.024	[0.020, 0.116]	**0.005**	0.146	0.034	[0.079, 0.213]	**<.001**	-0.019	0.026	[-0.071, 0.033]	0.472
Concurrent covariance												
Grade 3	0.041	0.030	[-0.018, 0.101]	0.175	0.057	0.043	[-0.027, 0.141]	0.186	0.039	0.038	[-0.036, 0.113]	0.310
Grade 4	0.054	0.023	[0.010, 0.098]	**0.016**	0.087	0.031	[0.027, 0.148]	**0.005**	0.026	0.025	[-0.022, 0.074]	0.295
Grade 5	0.060	0.025	[0.011, 0.108]	**0.015**	0.097	0.034	[0.031, 0.163]	**0.004**	0.029	0.027	[-0.025, 0.083]	0.296
Between-person correlation	0.258	0.039	[0.183, 0.334]	**<.001**	0.183	0.036	[0.112, 0.253]	**<.001**	0.255	0.044	[0.169, 0.342]	**<.001**

Note. Standardized estimates reported.

Table S7. RI-CLPMs on Depressive Symptoms and Externalizing Behaviors

	Full sample				Lower-income				Relatively higher-income			
Path	*β*	*SE*	95% CI	*p-value*	*β*	*SE*	95% CI	*p-value*	*β*	*SE*	95% CI	*p-*value
Autoregressive Paths												
Depressive symptoms												
Grade 3 → Grade 4	0.063	0.059	[-0.053, 0.179]	0.285	0.080	0.047	[-0.013, 0.173]	0.090	0.081	0.049	[-0.015, 0.177]	0.098
Grade 4 → Grade 5	0.065	0.065	[-0.063, 0.193]	0.320	0.082	0.053	[-0.022, 0.186]	0.122	0.085	0.056	[-0.024, 0.194]	0.125
Externalizing behavior												
Grade 3 → Grade 4	0.134	0.036	[0.064, 0.204]	**<.001**	0.136	0.034	[0.069, 0.203]	**<.001**	0.132	0.033	[0.067, 0.197]	**<.001**
Grade 4 → Grade 5	0.132	0.039	[0.055, 0.209]	**0.001**	0.131	0.037	[0.059, 0.203]	**<.001**	0.133	0.037	[0.061, 0.206]	**<.001**
Cross-lagged Paths												
Depressive symptoms to Externalizing												
Grade 3 → Grade 4	0.050	0.027	[-0.002, 0.102]	0.061	0.042	0.025	[-0.008, 0.092]	0.097	0.035	0.021	[-0.007, 0.077]	0.099
Grade 4 → Grade 5	0.051	0.028	[-0.003, 0.106]	0.066	0.044	0.027	[-0.008, 0.096]	0.099	0.037	0.023	[-0.008, 0.081]	0.104
Externalizing to Depressive symptoms												
Grade 3 → Grade 4	0.095	0.031	[0.033, 0.156]	**0.003**	0.061	0.023	[0.017, 0.106]	**0.007**	0.072	0.026	[0.021, 0.124]	**0.006**
Grade 4 → Grade 5	0.093	0.030	[0.033, 0.152]	**0.002**	0.059	0.022	[0.017, 0.101]	**0.006**	0.074	0.027	[0.022, 0.126]	**0.006**
Concurrent covariance												
Grade 3	0.054	0.030	[-0.005, 0.113]	0.072	0.057	0.045	[-0.030, 0.145]	0.198	0.040	0.031	[-0.021, 0.101]	0.203
Grade 4	0.049	0.027	[-0.004, 0.102]	0.069	0.018	0.015	[-0.012, 0.047]	0.236	0.028	0.024	[-0.018, 0.075]	0.236
Grade 5	0.051	0.028	[-0.004, 0.105]	0.072	0.018	0.015	[-0.012, 0.048]	0.239	0.030	0.026	[-0.020, 0.080]	0.236
Between-person correlation	0.146	0.035	[0.078, 0.214]	**<.001**	0.084	0.035	[0.015, 0.152]	**0.017**	0.133	0.049	[0.036, 0.229]	**0.007**

Note. Standardized estimates reported.

Table S8. Sensitivity Analyses Restricted to Children with the Same Respondent across Grades 3–5

	Full sample (N = 5,094)				Lower-income (n=1,835)				Relatively higher-income (n=3,259)			
Path	*β*	*SE*	95% CI	*p-value*	*β*	*SE*	95% CI	*p-value*	*β*	*SE*	95% CI	*p-*value
**Cross-lagged Paths**												
Internalizing Behaviors												
Depressive symptoms to Internalizing												
Grade 3 → Grade 4	0.042	0.023	[−0.003, 0.088]	0.067	0.086	0.034	[0.019, 0.153]	**0.011**	-0.008	0.027	[-0.061, 0.046]	0.780
Grade 4 → Grade 5	0.047	0.026	[−0.003, 0.098]	0.066	0.099	0.038	[0.025, 0.174]	**0.008**	-0.008	0.030	[-0.066, 0.050]	0.781
Internalizing to Depressive symptoms												
Grade 3 → Grade 4	0.057	0.024	[0.010, 0.104]	**0.018**	0.124	0.036	[0.054, 0.194]	**0.001**	-0.017	0.025	[-0.066, 0.032]	0.494
Grade 4 → Grade 5	0.062	0.025	[0.013, 0.110]	**0.013**	0.135	0.036	[0.065, 0.204]	**< .001**	-0.019	0.027	[-0.072, 0.035]	0.497
Externalizing Behaviors												
Depressive symptoms to Externalizing												
Grade 3 → Grade 4	0.049	0.026	[-0.002 , 0.100]	0.059	0.047	0.026	[-0.004, 0.098]	0.074	0.037	0.021	[-0.003, 0.078]	0.072
Grade 4 → Grade 5	0.047	0.024	[0.000, 0.094]	0.050	0.048	0.027	[-0.005, 0.101]	0.075	0.039	0.023	[-0.005, 0.084]	0.080
Externalizing to Depressive symptoms												
Grade 3 → Grade 4	0.094	0.032	[0.032, 0.156]	**0.003**	0.066	0.023	[0.021, 0.111]	**0.004**	0.078	0.027	[0.025, 0.131]	**0.004**
Grade 4 → Grade 5	0.095	0.032	[0.033, 0.156]	**0.003**	0.065	0.023	[0.021, 0.109]	**0.004**	0.081	0.028	[0.027, 0.135]	**0.003**

Note. The sensitivity analyses used the same RI-CLPM specification, equality-constraint procedure, MLR estimation, sampling weight, stratification, and clustering adjustments as the primary analyses. Standardized estimates reported. Only cross-lagged paths presented.

For internalizing behaviors, the fully constrained model was retained for the full sample, $\chi^2(6) = 5.22$, CFI = 1.000, TLI = 1.001, SRMR = .010, RMSEA = .000, 90% CI [.000, .017]. In the multiple-group analysis, the model in which the concurrent and cross-lagged paths were freely estimated was retained, $\chi^2(15) = 12.29$, CFI = 1.000, TLI = 1.002, SRMR = .015, RMSEA = .000, 90% CI [.000, .015]. For externalizing behaviors, the fully constrained model was retained for the full sample, $\chi^2(6) = 6.15$, CFI = 1.000, TLI = 1.000, SRMR = .013, RMSEA = .002, 90% CI [.000, .018]. In the multiple-group analysis, the fully constrained model was retained, $\chi^2(18) = 19.05$, CFI = 1.000, TLI = 1.000, SRMR = .022, RMSEA = .005, 90% CI [.000, .019].

Table S9. Sensitivity Analyses Using 100% of the Poverty Threshold

	Poverty group (n =898)				Non-poverty group (n= 4,422)			
Path	*β*	*SE*	95% CI	*p*-value	*β*	*SE*	95% CI	*p*-value
**Cross-lagged Paths**								
Internalizing Behaviors								
Depressive symptoms to Internalizing								
Grade 3 → Grade 4	0.065	0.032	[0.002, 0.128]	**0.042**	0.016	0.024	[-0.030, 0.062]	0.493
Grade 4 → Grade 5	0.078	0.034	[0.011, 0.145]	**0.022**	0.018	0.026	[-0.025, 0.068]	0.494
Internalizing to Depressive symptoms								
Grade 3 → Grade 4	0.150	0.051	[0.050, 0.250]	**0.003**	0.015	0.022	[-0.029, 0.059]	0.496
Grade 4 → Grade 5	0.150	0.048	[0.065, 0.244]	**0.002**	0.017	0.025	[-0.031, 0.065]	0.493
Externalizing Behaviors								
Depressive symptoms to Externalizing								
Grade 3 → Grade 4	0.052	0.028	[-0.002, 0.106]	0.061	0.048	0.025	[-0.001, 0.097]	0.056
Grade 4 → Grade 5	0.056	0.030	[-0.002, 0.114]	0.060	0.050	0.027	[-0.003, 0.103]	0.064
Externalizing to Depressive symptoms								
Grade 3 → Grade 4	0.077	0.024	[0.029, 0.124]	**0.001**	0.085	0.026	[0.035, 0.136]	**.001**
Grade 4 → Grade 5	0.068	0.022	[0.026, 0.110]	**0.002**	0.089	0.027	[0.036, 0.142]	**.001**

Note. The sensitivity analyses used the same RI-CLPM specification, equality-constraint procedure, MLR estimation, sampling weight, stratification, and clustering adjustments as the primary analyses. Standardized estimates reported. Only cross-lagged paths presented. For internalizing behaviors, the cross-lagged-free model was retained, $\chi^2(16) = 11.07$, CFI = 1.000, TLI = 1.004, SRMR = .014, RMSEA = .000, 90% CI [.000, .012]. For externalizing behaviors, the fully constrained model was retained, $\chi^2(18) = 16.58$, CFI = 1.000, TLI = 1.001, SRMR = .015, RMSEA = .000, 90% CI [.000, .016].
